# Abstract of the Proceedings of the Buffalo Medical Association

**Published:** 1857-12

**Authors:** Austin W. Nichols


ART. III.—*Abstract of the Proceedings of the Buffalo Medical Association.*

TUESDAY EVENING, Nov. 3, 1857.

The association met.

Present—Drs. Wyckoff, Newman, Strong, Miner, Root, A. Flint, Jr., Lemon, and A. W. Nichols.

The President being absent, the Vice-president, Dr. Wyckoff, took the chair.

On motion, the reading of the minutes of the last meeting was dispensed with.

Dr. A. W. Nichols was appointed secretary pro. tem.

*Dr. Root* stated, in connection with the report at a previous meeting of the association, on swallowing of foreign bodies, the circumstance of a person who had swallowed several false teeth attached to a metallic plate, whilst asleep; cannot say whether the foreign body was passed.

*Dr. A. Flint, Jr.*, related the following case of extensive injury to forearm:

The patient was a German boy, aged about 10 years, who had his left forearm crushed by the cars, while endeavoring to drive some cattle from the track. I saw the patient, a few hours after the accident, with Dr. Gay and Prof. Hamilton; the car had evidently passed obliquely over the arm, or one side of the arm, and had produced a fearful wound, carrying off bodily about the middle third of the radius; lacerating and contusing the muscles, and denuding the entire forearm of integument, which was, however, attached above at the elbow, and below at the wrist. None of the important vessels or nerves were injured; the joint at the wrist and elbow were uninjured, and the ulna was still sound. After a careful consideration and examination of the case, it was thought best not to operate, but to dress it as well as possible, and leave the rest to nature. The skin was accordingly tacked in its place as well as possible, and the arm laid on a board, with directions to keep the parts wet with cold water.

I saw the case about ten days after, and found that the integument had sloughed, leaving the entire forearm denuded; nearly all the rest of the radius had also come away. The surface was granulating and discharging healthy matter. The patient was in good spirits, and there were no signs of failure of the system in consequence of the immense drain. I regret that Dr. Gay is not present, who would be able to give a much fuller account of the case.

About two months after the injury I was passing the house and stopped to see the patient, but was told that he had removed to Hamburgh about a week before, and that at the time of his removal he was walking about, and the arm was doing well. I have heard nothing further from the case.

I mention this case as showing the conservatism of the present views of surgery in relation to injuries. I have no doubt but that a few years ago this limb would have inevitably been sacrificed, and must say that even now I have some doubts as to the expediency of allowing it to remain; the question is, will this immense surface ever be covered over. Prof. Hamilton suggested, that in the event of a failure we might shorten the arm by removing a portion of the ulna and thus diminish the extent of surface to be covered by new skin. It certainly would take a great length of time for such an immense ulcer to be filled up, if it ever could be. Though the result of this case is not able to be known, we learn an important lession in regard to the immediate danger of such an injury if the operation be deferred. When I saw the case the skin appeared to have escaped with comparatively little injury; and we had strong hopes of saving the greater portion of it. I would suggest that the probable cause of the death of the integument was the rupture of the vessels which came from thhe subjacent tissues, and that the proper vessels of the skin which were supplied from the attachment to the elbow and wrist, was not sufficient to maintain its vitality.

*Dr. Lemon* related a somewhat parallel case, in Dr. Newman's practice

And the association then adjourned.

AUSTIN W. NICHOLS, M. D.,
Secretary *pro tem.*

